# Porous Metal–Organic Framework Nanoparticles

**DOI:** 10.3390/nano12030527

**Published:** 2022-02-03

**Authors:** Juan M. Casas-Solvas, Antonio Vargas-Berenguel

**Affiliations:** Department of Chemistry and Physics, University of Almeria, Carretera Sacramento s/n, E-04120 Almeria, Spain; jmcasas@ual.es (J.M.C.-S.); avargas@ual.es (A.V.-B.)

Metal–organic frameworks (MOFs) are hybrid crystalline particles composed of metal cations and organic linkers. Ranging from micro- to nanoscale depending on the preparation conditions, they have achieved a prevalent position among porous materials. The fact that varying either the metal cation or the organic component leads to a wide range of pore sizes and structures has made them very appealing materials in a broad variety of fields, including gas storage, heterogeneus catalysis, separation, imaging, biosensing, agriculture, and biomedicine. 

By optimizing the internal pore volume, many molecules of different natures can be accommodated within the matrix. For instance, the anticancer drug doxorubicin is well known to enter within iron trimesate MIL-100(Fe) nanoMOF. However, the use of this inclusion complex in biomedicine requires the controlled release of the drug. As reported in one of the articles within this Special Issue [1], this goal can be achieved either by modifying the way the drug is loaded into the MOF or by noncovalently coating the surface with appropriate biocompatible materials. Furthermore, the latter can also lead to a higher colloidal stability of the particles. The innovative use of the ssNMR technique on these inclusion complexes associated with a selective isotope labeling strategy gave the authors deeper insights into both the structure of the complexes as well as to the drug release rates and mechanism.

The use of nanoMOFs in the field of biomedicine also requires a good deal of knowledge about the degradation mechanisms of these materials. For instance, MIL-100(Fe) can cause an increased iron concentration in both the liver and spleen, but these levels remain far from being toxic to these organs. Furthermore, urine and feces excrete excess iron after 15 days. Understanding how the structural defects of the particles affect their degradation rate is of pivotal importance to pave the way for their clinical use as delivery systems in biological media. One of the articles within this Special Issue describes the latest findings on this issue through the use of atomic force microscopy [2].

Agriculture is another field where the use of nanoMOFs is of great interest. In the third article, the inclusion of the fungicide diniconazole into a MIL-101(Fe) nanoMOF containing free NH_2_ groups within its matrix is described [3]. Furthermore, the subsequent modification of the inclusion complex with polydopamine allowed a controlled release of the cargo in response to the external pH of the media. This novel material has the potential to help sustainably protect crops against Fusarium head blight.

The aim of this Special Issue is to offer the readers a compilation of cutting-edge research within the porous metal–organic framework nanoparticles field, providing insights into its current challenges and future directions and further increasing interest of this topic. We hope that all readers will enjoy it.

## References

[1] Li X., Porcino M., Qiu J., Constantin D., Martineau-Corcos C., Gref R. (2021). Doxorubicin-loaded metal-organic frameworks nanoparticles with engineered cyclodextrin coatings: Insights on drug location by solid state NMR spectroscopy. Nanomaterials.

[2] Christodoulou I., Bourguignon T., Li X., Patriarche G., Serre C., Marlière C., Gref R. (2021). Degradation mechanism of porous metal-organic frameworks by in situ atomic force microscopy. Nanomaterials.

[3] Shan Y., Xu C., Zhang H., Chen H., Bilal M., Niu S., Cao L., Huang Q. (2020). Polydopamine-modified metal–organic frameworks, NH_2_-Fe-MIL-101, as pH-sensitive nanocarriers for controlled pesticide release. Nanomaterials.

